# Nonhuman Primate Adenoviruses of the Human Adenovirus B Species Are Potent and Broadly Acting Oncolytic Vector Candidates

**DOI:** 10.1089/hum.2021.216

**Published:** 2022-03-16

**Authors:** Selas T.F. Bots, Vera Kemp, Steve J. Cramer, Diana J.M. van den Wollenberg, Marten Hornsveld, Martine L.M. Lamfers, Gabri van der Pluijm, Rob C. Hoeben

**Affiliations:** ^1^ Department of Cell and Chemical Biology, Leiden University Medical Center, Leiden, the Netherlands; ^2^ Department of Neurosurgery, Brain Tumor Center, Erasmus Medical Center, Rotterdam, the Netherlands.; ^3^ Department of Urology, Leiden University Medical Center, Leiden, the Netherlands

**Keywords:** oncolytic virus therapy, adenovirus, cancer, nonhuman primate adenovirus

## Abstract

The use of human adenoviruses (hAds) as oncolytic agents has demonstrated considerable potential. However, their efficacy in clinical studies is generally moderate and often varies between patients. This may, in part, be attributable to variable pre-existing neutralizing immunity in patients, which can impact the antitumor efficacy and lead to response heterogeneity. Our aim was to isolate new Ads for the development of oncolytic vectors with low prevalence of neutralizing immunity in the human population. To this end, we isolated a collection of new nonhuman primate (nhp) Ads from stool samples of four great ape species held captive. We elected 12 isolates comprising the broadest genetic variability for further characterization. For three new nhpAds, all classified as the human adenovirus B (HAdV-B) species, no neutralizing activity could be detected when exposed to a preparation of immunoglobulins isolated from a pool of >1,000 donors as a surrogate of population immunity. In addition, the nhpAds of the HAdV-B species showed enhanced oncolytic potency compared to nhpAds of the HAdV-C species as well as to human adenovirus type 5 (HAdV-C5) *in vitro* when tested in a panel of 29 human cancer cell lines. Next-generation sequencing of the viral genomes revealed higher sequence similarity between hAds and nhpAds of HAdV-B compared to HAdV-C, which might underlie the differences in oncolytic ability. As a proof-of-concept, the Rb-binding domain of the E1A protein of the gorilla-derived HAdV-B nhpAd-lumc007 was deleted, thereby creating a new oncolytic derivative, which demonstrated increased oncolytic potential compared to HAdV-C5. Collectively, our data demonstrate that nhpAds of the HAdV-B species can serve as an alternative for the development of potent oncolytic Ad vectors with limited pre-existing neutralizing immunity in humans.

## INTRODUCTION

Advanced-stage cancer patients are currently subjected to surgery or stringent regimens of chemotherapy and radiation therapy. While effective in some cases, in others, life expectancy is marginally increased and the quality of life reduced by such therapies.^1,2^ Therefore, more efficacious therapies are much needed.

Oncolytic viruses (OVs) have emerged as a new therapeutic option in cancer treatment. The viruses, by virtue of their preference to infect, replicate in, and subsequently kill cancerous cells as opposed to normal cells, can provide an inherent cancer cell selectivity. Human adenoviruses (hAds) are one of the leading candidates for the development of OVs and have demonstrated the potential to induce immunogenic cell death,^3^ often leading to an immune response directed against the tumor cells.^4,5^ In addition, they exhibit an excellent safety profile.^6,7^ The hAds are nonenveloped viruses with a linear double-stranded DNA genome of 34–37 kB in size.

In humans, over one hundred different “types” have been recognized based on serology and genome sequencing.^8,9^ These types are classified into different “species” (formerly “subgroups”) named human adenovirus (HAdV) A through G.^10^ Most of the current adenoviral vectors are based on HAdV-C type 5 (HAdV-C5), which is also one of the most prevalent types circulating in the human population.^11^ As oncolytic agents, Ads based on HAdV-C5 have so far demonstrated the safety of the approach,^7,12^ but the clinical results are often moderate and variable with marked patient-to-patient variation.^13^ It is possible that pre-existing neutralizing immunity against HAdV-C5 may underly, in part, the response heterogeneity.

In vaccine development, alternative hAd types with low seroprevalence have been exploited to circumvent pre-existing neutralizing immunity like HAdV-D26, HAdV-B35, HAdV-D48,^11,14–16^ and HAdV-D19a/64,^17^ as well as vectors derived from nonhuman primate Ads (nhpAds).^18^ nhpAds are of interest, especially as they do not circulate in the human population, and as such, there may be a reduced frequency of pre-existing neutralizing immunity in humans. Moreover, nhpAds demonstrate high sequence similarity to hAds and share many characteristics, like receptor use and modulation of immune responses.^19^ In fact, there is clear evidence that there have been several host-species switches from apes to humans over the course of evolution.^20^

Following the successful use of a chimpanzee Ad vector in coronavirus disease 2019 (COVID-19) vaccination,^21^ nhpAds may also be exploited as replicating OVs in humans.^19^ However, Ad replication is generally restricted to a single host species and the potential of these nhpAds for use as oncolytic agents is yet inconclusive. Therefore, we sought to carefully address whether nhpAds have the potential to be used for the generation of oncolytic Ad vectors with low seroprevalence in the human population.

In this study, we isolated new nhpAds from several great ape species and characterized 12 isolates, which are grouped in either the HAdV-B or HAdV-C species for their potential as oncolytic agents in human cells. In contrast to the nhpAds of HAdV-C, the nhpAds of HAdV-B were not neutralized by pooled human immunoglobulins. Moreover, in a panel of 29 human cancer-associated cell lines, two out of three nhpAds classified as HAdV-B demonstrated stronger lytic activity and infected a broader range of cancer cells in comparison to nhpAds of HAdV-C and HAdV-C5. As a proof of concept, the HAdV-B candidate AdV-lumc007 was cloned into a plasmid vector and modified by deletion of one of the E1A Rb-binding domains as to constrain its replication capacity in normal cells. As a result, the new gorilla-derived oncolytic Ad AdV-lumc007ΔRbD or “Goravir” was generated as a fast-acting and broadly applicable OV with low seroprevalence in humans.

## MATERIALS AND METHODS

### Cell lines

Cells were cultured in high-glucose Dulbecco's modified Eagle's medium (DMEM; Gibco, Massachusetts) supplemented with 8% fetal bovine serum (FBS; Invitrogen, California) and 1% Penicillin-Streptomycin (P/S; Gibco), unless otherwise specified. The HAdV-C5 E1-transformed human embryonic retinoblast cell line HER911 has previously been designed for the production of replication-incompetent adenoviral vectors.^22^ The human lung carcinoma cell line A549 (CCL-185) was purchased from the American Type Culture Collection (ATCC, Virginia). VH10, a normal foreskin fibroblast cell line, was kindly provided by Binie Klein.^23^ The normal human fetal long fibroblast MRC-5 (AG05965-C) cells were purchased from the Coriell Institute (New Jersey). The patient-derived glioblastoma stem cell (GSC) lines GS343peri, GS304, GS203, GS281, GS324core, GS245, GS186core, GS452, GS365, and GS436 have been described elsewhere^24^ and were cultured in DMEM–F12 (Gibco) with 1% P/S, B27 (Invitrogen), human epidermal growth factor (5 mg/mL; Tebu-Bio, Heerhugowaard, the Netherlands), human basic fibroblast growth factor (5 mg/mL; Tebu-Bio), and heparin (5 mg/mL; Sigma-Aldrich, Missouri). GSCs were cultured on dishes coated with growth factor-reduced extracellular matrix (BD Biosciences, California). Fresh glioma tissue samples were obtained directly from the operating room of the Erasmus Medical Center, the Netherlands. The use of patient tissue for this study was approved by the local ethics committee and all patients signed an informed consent form according to the guidelines of the Institutional Review Board. The study was conducted according to the guidelines of the Declaration of Helsinki, ethical statement was obtained from the Institutional Review Board—Medical Ethical Review Committee Erasmus MC, code MEC-2013-090, on 22 April 2013.

Urinary bladder carcinoma cell lines 5637 (HTB-9; ATCC) and TCC-SUP (HTB-5; ATCC) were cultured in Eagle's minimal essential medium (EMEM; Gibco) supplemented with 10% FBS and 1% P/S. The UM-UC-3Luc2 cell line was cultured in EMEM supplemented with 10% FBS, 800 μg/mL G-418 (LifeTechnologies), and 1% P/S, and is described elsewhere.^25^ The HCV29 bladder carcinoma cell line (kind gift from dr. L.A. Dyrskjot, Aarhus, Denmark) was cultured in RPMI-1640 medium supplemented with 10% FBS and 1% P/S. RT-4 urinary bladder cancer cells (ATTC, HTB-2) were cultured in McCoy's 5A medium (Gibco) supplemented with 10% FBS and 1% P/S. Urinary bladder cancer cell line J82 (HTB-1) was purchased from the ATCC. The urinary bladder carcinoma T24 (HTB-4; ATCC) and the prostate cancer cell lines 22RVI (kind gift from prof B. Watson, Dublin, Ireland), C4–2B4 (kind gift from prof G. Thalmann, Berne, Switzerland), Du145 (ATTC, HTB-81), and PnT2C2 (kind gift from prof N.J. Maitland, York UK) were cultured in RPMI-1640 medium (Biowest, Nuaillé, France) supplemented with 10% FBS, 1% P/S, and 1 × GlutaMAX™ supplement (Gibco). The prostate cancer cell line PC-3M-Pro4Luc2 was cultured in DMEM supplemented with 10% HyClone TM Fetal Clone^®^ II (Thermo Fisher Scientific, Massachusetts, United States), 800 μg/mL G-418, and 1% P/S, and is described elsewhere.^26^ The pancreatic cancer cell lines PatuS and PatuT were obtained from the DSMZ culture bank (Braunschweig, Germany). The pancreatic stellate cell line RLT-PSC was a kind gift from prof M. Löhr (Stockholm, Sweden) and is described elsewhere.^27^ The pancreatic cancer cell lines BxPC-3 (CRL-1687), HPAF-II (CRL-1997), Mia-PaCa2 (CRM-CRL-1420), and PANC-1 (CRL-1469) were all purchased from the ATCC. All cells were cultured in an atmosphere of 5% CO_2_ at 37°C.

### Isolation of new nhpAd isolates

Fecal samples were obtained from *Pan troglodytes* (chimpanzee), *Pan paniscus* (bonobo), *Gorilla gorilla gorilla* (Western Lowland gorilla), and *Pongo pygmaeus* (Bornean orangutan), held in captivity in Dutch zoos. Viruses were isolated essentially as described by Roy *et al*.^28^ Fecal aliquots (250–500 mg) were resuspended in 5 mL phosphate-buffered saline without Ca^2+^ and Mg^2+^ (PBS^–^) by thorough mixing (3 × 20 s) on a vortex mixer. The suspension was cleared by centrifugation for 5 min in a tabletop centrifuge at 6,000 *g*. From the cleared suspension, 4 mL was isolated and passed twice through 0.45 μm Acrodisc^®^ Syringe Filters with an HT Tuffryn^®^ membrane (PN4148; Pall Life Sciences, New York). From each of the filtrates, 100 and 10 μL aliquots were added to cultures of HER911 cells grown in ø six-well plates (Thermo Scientific) in DMEM supplemented with 8% FBS, 1% P/S, gentamicin (200 μg/mL; Gibco), and amphotericin B (2 μg/mL; Gibco).

The cultures were inspected every other day for signs of cytopathic effects (CPEs). When >10% of the cells exhibited marked CPE, cells were harvested by flushing the cells from the dish with medium and collected in a 10 mL polypropylene tube (Greiner Bio-One, Kremsmünster, Austria). Cells in the medium were lysed by freeze-thawing (F/T) three times, after which the cell debris was pelleted by centrifugation in a tabletop centrifuge for 3 min at 6,000 *g*. From the supernatant, 200 μL was added to a fresh near-confluent culture of HER911 cells grown on ø 10 cm dish (Greiner Bio-One) in DMEM supplemented with 8% FBS, 1% P/S, gentamicin (200 μg/mL), and amphotericin B (2 μg/mL). When CPE was nearly complete, the medium with the cells was collected and the cells were lysed by three cycles of F/T. The lysates were cleared by centrifugation in a tabletop centrifuge for 3 min at 6,000 *g*. The lysates were stored at -20°C.

#### *Hexon* hypervariable region sequencing

Near confluent cultures of HER911 cells in ø 6 cm dishes were exposed to 100 μL of the virus-containing supernatant. Upon marked CPE, cells were collected in the medium and subjected to the HIRT extraction procedure optimized for Ad DNA isolation. Cells were pelleted by centrifugation for 5 min at 1,500 *g*. The pellet was washed once in PBS and subsequently 600 μL lysis mix (10 mM Tris-HCl pH 7.5, 10 mM EDTA, and 0.6% SDS) was added. The cell pellet was gently resuspended and 150 μL 5M NaCl was added. Samples were incubated at 4°C overnight. The next day, samples were centrifuged at 10,000 rpm for 50 min at 4°C. To the supernatant, 600 μL isopropanol was added and after gentle mixing, the tubes were kept a room temperature (RT) for 15 min. Next, samples were spun at 16,000 *g* for 30 min at RT. The pellet was resuspended in 40 μL 10 mM Tris and 1 mM EDTA with RNase (50 μg RNase/mL) and incubated at 37°C for 20 min. Samples were treated with proteinase K, and cleaned by phenol/chloroform extraction according to standard techniques. DNA concentrations were determined by NanoDrop™ 1000 Spectrophotometer (Thermo Scientific).

Approximately 10 ng of DNA was used for PCR amplification of the *hexon* hypervariable region (HHVR) 1–7 using the following primers: 5′-CAGGATGCTTCGGAGTACCTGAG-3′ (forward primer) and 5′-TTGGCNGGDATDGGGTAVAGCATGTT-3′ (reverse primer), in which the “N” is used to indicate any base, “D” indicates A, G, or T, and “V” denotes A, C, or G. A standard PCR was performed (30 s at 55°C, 1 min at 72°C, and 1 min at 95°C, 30 cycles) and the sequences were determined by Sanger sequencing.

### nhpAd isolate propagation and titration

For each of the elected virus isolates, a T-25 flask (Greiner Bio-One) was seeded with HER911 cells and grown to near confluency. Next, cells were infected with 100 μL virus in DMEM supplemented with 2% FBS and 1% P/S. Upon complete CPE, cell cultures were collected and F/T for one cycle, after which the cell debris was pelleted by centrifugation in a tabletop centrifuge for 5 min at 1,500 *g*. The virus-containing supernatants were collected and stored at −20°C. This propagation procedure was repeated using T-75 flasks (Greiner Bio-One) and either 100 or 500 μL of the previously generated virus batches, depending on the timeframe in which CPE was present in those T-25 culture flasks. Within 1–3 days, all cultures demonstrated complete CPE and were collected as described above. Samples were F/T for four cycles before pelleting the cell debris and collecting the supernatant. Virus supernatants were aliquoted and stored at −20°C. Virus titers were determined by standard plaque assay using the HER911 cell line.

### Neutralization assay

One day before titration, HER911 cells were seeded in 96-well flat-bottom tissue culture plates (Greiner Bio-One) in DMEM supplemented with 8% FBS. On the day of infection, a twofold dilution series of Nanogam^®^ (Sanquin, Amsterdam, Netherlands) was prepared starting at 2.5 mg/mL (1:4) in DMEM supplemented with 2% horse serum (HS). Each dilution was mixed with 100 plaque-forming units (PFU) of virus in a 1:1 volume and incubated for 45 min at 37°C to allow the antibodies to bind. Virus without Nanogam was used as a control. The culture medium was removed from HER911 cells and replaced by 100 μL intravenous immunoglobulin (IVIg):virus dilution in DMEM supplemented with 2% FBS and 1% P/S. Cell viability was determined at 7 days post-infection by crystal violet according to manufacturer's protocol. The IVIg dilution range for complete neutralization of the virus was based on three biologically independent experiments.

### Oncolysis assay

One day before infection, cells were seeded at 5,000 cells/well in a 96-well plate in the appropriate cell culture medium and incubated o/n at an atmosphere of 5% CO_2_ at 37°C. Next, cells were infected at the indicated multiplicity of infection (MOI) in cell culture medium supplemented with 2% serum if the respective cell line was cultured in the presence of serum under normal growth conditions; otherwise, standard culture medium was used. Cell viability was measured either at fixed time points by cell proliferation reagent kit WST-1 (Merck, New Jersey) according to manufacturer's instructions or continuously every 8 hours by use of the Incucyte Cytotox Red Dye Reagent (4632; Satorius, Noordwijk, The Netherlands) and the Incucyte S3 Live-Cell Analysis System (Satorius).

### Next-generation sequencing

DNA from virus-infected cells isolated by HIRT extraction was analyzed by sequencing on an Illumina platform by BaseClear B.V. (Leiden, the Netherlands). The viral genomes were assembled *de novo* and viral genes were annotated by comparing the DNA sequences with the Ad genomes annotated in the Genbank nucleotide databases at NCBI (https://www.ncbi.nlm.nih.gov/nuccore/). All nhpAd full genome sequences are available in Genbank (Table 1).

**Table 1.** Characteristics of the 12 nhpAd genomes covering the broadest genetic diversity in the hexon hypervariable region

Isolate	Host	Species	Genome Length (bp)	Accession Number
AdV-lumc001	*Pan paniscus*	HAdV-C	37,820	MZ882379
AdV-lumc002	*Pan paniscus*	HAdV-C	37,702	MZ882389
AdV-lumc003	*Pan paniscus*	HAdV-C	37,724	MZ882380
AdV-lumc004	*Pan paniscus*	HAdV-C	37,647	MZ882381
AdV-lumc005	*Pongo pygmaeus*	HAdV-C	37,034	MZ882382
AdV-lumc006	*Gorilla gorilla gorilla*	HAdV-B	35,179	MZ882387
AdV-lumc007	*Gorilla gorilla gorilla*	HAdV-B	35,605	MZ882390
AdV-lumc008	*Gorilla gorilla gorilla*	HAdV-C	37,200	MZ882383
AdV-lumc009	*Pan troglodytes*	HAdV-C	37,885	MZ882384
AdV-lumc010	*Pan troglodytes*	HAdV-C	37,738	MZ882385
AdV-lumc011	*Pan troglodytes*	HAdV-C	37,780	MZ882386
AdV-lumc012	*Pan troglodytes*	HAdV-B	35,597	MZ882388

### Construction of the AdV-lumc007 vector system

A synthetic double-stranded DNA fragment was synthesized by Eurofins (Ebersberg, Germany) that encompassed the left-hand side nucleotides no. 1–513 and right-hand side nucleotides no. 34913–35605 of AdV-lumc007, which were separated by the sequence 5′-GATATCGAGGTTAAC-3′ to provide unique *Eco*RV and *Hpa*I restriction sites. The entire fragment was flanked on either side by the sequence 5′-ACGCGTATTTAAAT-3′ to generate unique *Mlu*I and *Swa*I restriction sites. The fragment was inserted into the low-copy number plasmid pACNR1181.^29^ Next, *Escherichia coli* bacterial cells (BJ5183) were transformed with the generated plasmid after digestion with *Eco*RV and *Hpa*I, as well as the AdV-lumc007 DNA genome previously isolated by HIRT extraction in a 1:1 molecular ratio according to methods described elsewhere.^30,31^ Colonies that arose after ampicillin selection were screened for the anticipated restriction pattern by digestion with *Hin*cII, from which one was selected.

For production of Ad particles, the newly generated plasmid containing the full AdV-lumc007 genome was released from the plasmid by digestion with *Swa*I. HER911 cells were transfected with 1.5 μg plasmid using 3 μL polyethyleneimine (PEI, 1.0 mg/mL, cat. 23966; Polysciences, Hirschberg an der Bergstrasse, Germany) per μg DNA. Three days post-transfection, the cells were harvested and lysed by one cycle of F/T. After pelleting the cell debris, the cleared supernatant containing the Ad was passed through a 0.45 μm Acrodisc^©^ Syringe filter (PN4148, PALL Life Sciences). The filtered supernatant was aliquoted and stored at −20°C until further use.

#### CsCl purification of Ads

HER911 cells were seeded to confluency in T-75 flasks and infected with Ad at MOI 2. Upon complete CPE, cells were harvested and centrifuged at 300 *g* for 7 min. Supernatant was discarded and the cell pellet was taken up in 2 mL PBS with Ca^2+^ and Mg^2+^ (PBS^++^; Fresenius Kabi GmbH, Graz, Austria) supplemented with 2% HS. Virus was released from the cells by three cycles of F/T and cell debris was removed from the Ad-containing lysate by centrifugation at 700 *g* for 5 min. A cesium chloride-purified batch of Ad was prepared by loading the cleared freeze-thaw lysates onto a discontinuous CsCl gradient (1.45 and 1.20 g/cm^3^) in PBS. After centrifugation in a SW 41 Ti rotor (Beckman Coulter Life Sciences, Woerden, the Netherlands) at 30,000 rpm for 2 h at 16°C in an Optima XE-90 Ultracentrifuge (Beckman), the lower band, containing the infectious particles, was harvested and transferred to a continuous 1.33 g/cm^3^ CsCl gradient in PBS.

Another centrifugation round was performed in a Type 70.i Ti rotor (Beckman) at 48,000 rpm overnight at 16°C in an Optima XE-90 Ultracentrifuge. The remaining single band was isolated and desalted in an Amicon Ultra 100 K device (Millipore, Massachusetts) according to manufacturer's manual. The CsCl-purified Ad was recovered in Ad storage buffer (140 mM NaCl, 5.0 mM Na_2_HPO_4_·2H_2_O, 1.5 mM KH_2_PO_4_, pH 7.8, and 5% sucrose). The particle concentration was calculated based on the OD260 values before storage at −80°C.^32^ Viral titer was determined by standard TCID_50_ assay on HER911 cells.

### Determination of Ad genome copy numbers

A549 cells were infected with virus at MOI 2 in DMEM supplemented with 2% FBS and 1% P/S. Cells were collected at the indicated time point and prepared for genomic DNA (gDNA) isolation according to the mammalian cell lysate protocol of the Purelink™ Genomic DNA Kit (K182000; Invitrogen), and gDNA was isolated according to the manufacturer's instructions. DNA concentrations were determined by NanoDrop 1000. Approximately 1 ng DNA was used for qPCR amplification of a conserved region in DNA polymerase of human and nhpAds, using separate primers for the HAdV-B and HAdV-C species:

DNA Pol Species B (forward primer) 5′-ATGATGTCATAACCTGGTTGG-3′,

DNA Pol species B (reverse primer) 5′-TTACCGTGCAGACAAAGACG-3′,

DNA Pol species C (forward primer) 5′-ATGACCAGCATGAAGGGCA-3′, and

DNA Pol species C (reverse primer) 5′-CGGGAGATCCAGTTCTTCC-3′.

A standard qPCR program was followed: 3 min at 95°C (30 s at 95°C, 15 s at 60°C, and 30 s at 72°C × 35 cycles) and 10 min at 95°C. A standard curve was generated from a *Cla*I deletion mutant plasmid of AdV-lumc007 containing 9.629 bp of the left-hand terminus of the genome (species B) or the vector plasmid pMAd5, which contains bp 3,330 to bp 8,915 of HAdV-C5 (species C). Virus genome copy numbers were calculated as described previously.^33^

### Generation of the AdV-lumc007ΔRbD vector plasmid

Using a *Cla*I deletion mutant plasmid of AdV-lumc007 containing the first 9.629 bp, a 24-bp deletion was created removing bp 894 to 917 bp (ΔRbD) corresponding to the Rb-binding domain of the E1A protein, by *in vivo* assembly (IVA) cloning, as previously described.^34^ The PCR product was run on an agarose gel and a band of ∼10 kB was excised and purified using the QIAEX II Gel extraction kit (Qiagen) according to manufacturer's instructions. The mutant plasmid was introduced in *E. coli*, as described earlier. In the colonies that arose after ampicillin selection, the 24-bp deletion was confirmed by *Ear*I digestion, followed by Sanger sequencing. Both plasmids (*Cla*I-del mutant containing the ΔRbD mutation and complete wild-type AdV-lumc007 plasmid) were prepared by enzymatic digestion using *Swa*I and *Swa*I/*Swa*BI, respectively. The two plasmids were mixed 1:1 using a total of 1.5 μg DNA and added to HER911 cells with 3 μL PEI (1.0 mg/mL) per μg DNA.

Seven days post-transfection, cells were harvested and lysed by three cycles of F/T. After pelleting the cell debris, the cleared supernatant containing the Ad was used to infect a fresh culture of HER911 cells. At 4 days post-infection, complete CPE was observed and cells were harvested and lysed by three cycles of F/T. The Ad-containing supernatant was used to infect HER911 for plaque purification. After 2 days, several plaques arose, which were picked and added to fresh cultures of HER911 cells. Upon complete CPE, cells were harvested and lysed by freeze-thawing. After pelleting the cell debris, the cleared supernatant containing the plaque-purified Ad was passed through a 0.45 μm Acrodisc© Syringe filter.

Next, semiconfluent HER911 cells were seeded in a six-well plate and infected with 100 μL of the plaque-purified virus. After several days, supernatant was collected and added to fresh cultures of HER911 cells. This was repeated until complete CPE was observed at 2 days post-infection. Infected cells were collected and DNA was isolated using HIRT extraction and 50 ng of DNA was used for PCR amplification of the E1A Rb-binding domain using the following primers: 5′-TGAGACCCTTGATACCCCAGG-3′ (forward primer) and 5′-AGCAAAGCGAGCACAACAGT-3′ (reverse primer). A standard PCR program was followed (3 min at 95°C, [30 s at 95°C, 15 s at 60°C, and 30 s at 72°C] × 35 cycles) using an MJ Research PTC-200 Thermal Cycler (Bio-Rad) and half of the sample was run on an agarose gel. For the samples containing a DNA product at the expected height, the remainder of the sample was analyzed by Sanger sequencing to validate the deletion. The resulting virus AdV-lumc007ΔRbD was named “Goravir.”

## RESULTS

### Isolation and selection of nhpAds

To obtain new nhpAd isolates, nhpAds were isolated essentially as described by Roy *et al*.^28^ In short, stool samples were acquired from four great ape species held captive in Dutch zoos, including *Pan paniscus* (bonobo), *Pan troglodytes* (chimpanzee), *Gorilla gorilla gorilla* (Western Lowland gorilla), and *Pongo pygmaeus* (Bornean orangutan). Next, the fecal samples were suspended in PBS, cleared, and filtered before addition to cultures of the Ad producer cell line HER911. To isolate viruses with replication potential in human cells, HER911 cell cultures were examined for signs of viral infection by the presence of CPE.

From all 127 cultures, 52 demonstrated signs of CPE (40.9%), typically between 4 and 21 days post-infection (Supplementary Table S1). Upon completion, CPE-infected cells were collected and the freeze-thaw lysates were used to reinfect fresh cultures of HER911 cells, which were subjected to DNA isolation. A PCR was performed on the hypervariable region (HVR) of the hexon protein of human and nhpAds, and positive samples were sent for sequencing. A total of 42 unique HHVR sequences were identified, of which, the majority of viruses were classified as HAdV-C (70%), followed by HAdV-B and HAdV-E (each 15%, Supplementary Table S1). The HHVR is a primary target for neutralizing antibodies (nAb) and modifications in this region have been shown to circumvent pre-existing neutralizing immunity.^35,36^ Therefore, 12 isolates encompassing a broad genetic diversity of the HHVRs in our collection were elected for further characterization as oncolytic agents (Table 1).

Isolates grouped as HAdV-E were excluded as a precautionary measure as vaccination with HAdV-E4, the sole human-derived member of the HAdV-E species, was shown to cross-neutralize with other human and nhpAd types from several Ad species.^37^ Consequently, this could render the chances of pre-existing neutralizing immunity against these types higher and could hamper future translation of vector derivatives into the clinic. Therefore, only types grouped as HAdV-B or HAdV-C were included in the panel. The elected nhpAd isolates were attributed number 1 through 12, pending their official approval as new Ad types.

### No measurable pre-existing immunity against nhpAds of HAdV-B

The nhpAds do not circulate at a detectable frequency in the human population and as such, pre-existing neutralizing immunity to these viruses may be anticipated to be low. To confirm this, we tested the prevalence of nAbs against the panel of Ad isolates using Nanogam, a human intravenous immunoglobulin (IVIg) preparation of >1,000 healthy donors. This could serve as a surrogate for humoral immunity in the population (Table 2). Neutralization titers for complete neutralization of the HAdV-C nhpAds ranged from 1:8 to 1:128, which, in some cases, was considerably lower compared to HAdV-C5 (1:128–1:256). However, these data do suggest that there is considerable cross-neutralization between the HAdV-C nhpAds and antibodies directed against Ad types circulating in the human population.

**Table 2.** Anti-Ad neutralizing immunity against nhpAds

Adenovirus	Species	IVIg
HAdV-C5	HAdV-C	1:128–1:256
AdV-lumc001	HAdV-C	1:32–1:64
AdV-lumc002	HAdV-C	1:64–1:128
AdV-lumc003	HAdV-C	1:64–1:128
AdV-lumc004	HAdV-C	1:32–1:64
AdV-lumc005	HAdV-C	1:16–1:32
AdV-lumc006	HAdV-B	<1:8
AdV-lumc007	HAdV-B	<1:8
AdV-lumc008	HAdV-C	1:8–1:16
AdV-lumc009	HAdV-C	1:32–1:64
AdV-lumc010	HAdV-C	1:32–1:64
AdV-lumc011	HAdV-C	1:64–1:128
AdV-lumc012	HAdV-B	<1:8

IVIg, intravenous immunoglobulin.

For the nhpAds grouped as HAdV-B, the differences were more striking as the neutralizing titer for all three viruses (AdV-lumc006, AdV-lumc007, and AdV-lumc012) exceeded the highest concentration tested. The absence of neutralizing activity in the Nanogam preparation against the three HAdV-B nhpAds could indicate that there is no significant pre-existing neutralizing immunity in the Dutch population against the nhpAds of HAdV-B.

### nhpAds can kill transformed cells

**Figure 1.**
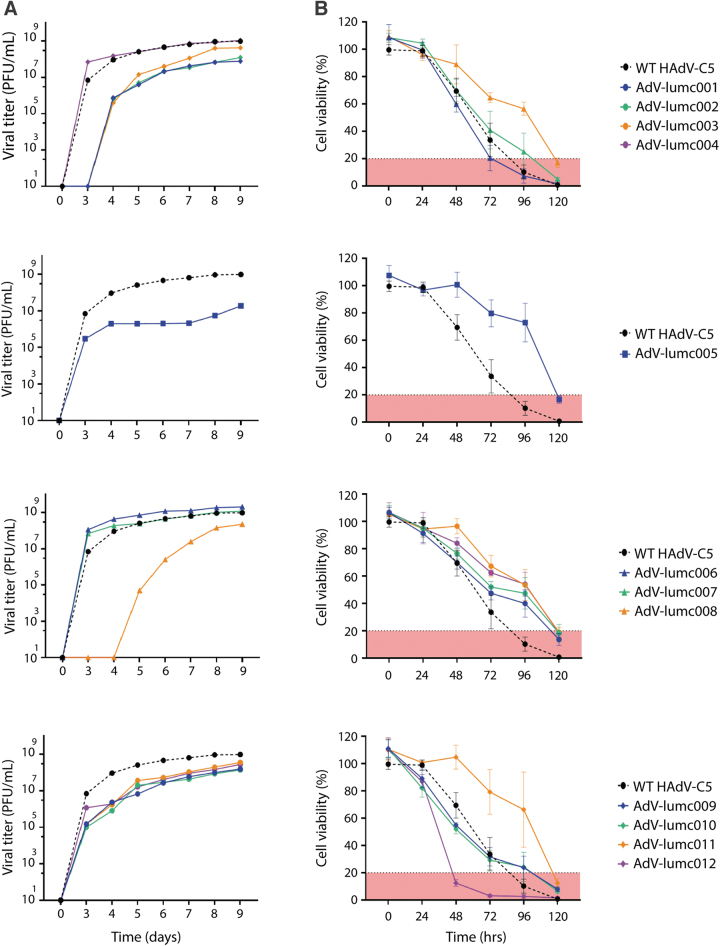
Viral replication and cell killing kinetics of nhpAds in human cells. Wild-type HAdV-C5 and nhpAd isolates derived from—*top* to *bottom panels*—bonobo, orangutan, gorilla, and chimpanzee were **(A)** propagated on HER911 cells and viral titers were determined by standard plaque assay. Plaques were counted daily over the course of 9 days and the estimated viral titer is represented for each isolate; **(B)** used to infect A549 cells at MOI 2 and cell viability was measured every 24 hours for 7 days. Mean cell viability ≤20% (*red* area) was considered a strong oncolytic effect. Mean and SD are depicted of *n* = 3. MOI, multiplicity of infection.

To verify that our selection of viruses was capable of efficient replication in human cells, all nhpAds isolates were first propagated on the HER911 cell line. A seed batch of virus was produced and cultures were collected upon complete CPE, generally between 2 and 6 days post-infection. From the seed batch, a second virus batch was generated for which all the viruses demonstrated complete CPE within 72 hours. The resulting viral titers were determined by standard plaque assay (Fig. 1A). All the viruses formed readily detectable plaques with kinetics similar to the development of plaques with HAdV-C5, which served as a reference control.

Of note, AdV-lumc005 represents the first report of an orangutan-derived replicating Ad. Final yields differed 100-fold between the different virus isolates (10^7^–10^9^ PFU/mL). Interestingly, this could not be directly attributed to their respective host species and might be due to the use of nonoptimized conditions for the production of initial stocks. Regardless, all virus isolates demonstrated productive infection of HER911 cells. Moreover, the resulting titers can probably be increased by optimization of the production procedure and would allow yields sufficient for clinical translation of these viruses.

The HER911 cell line contains a plasmid encompassing the left end of the HAdV-C5 genome, expressing the Ad early (*E*) *1A* and *E1B* genes, and is designed to facilitate the production of replication-deficient adenoviral particles.^22^ As the presence of these adenoviral genes could facilitate replication of the nhpAd isolates, virus-induced cell killing was assessed using the A549 human lung carcinoma cell line. Virus stocks were normalized according to infectious titers and at this stage, no effort was taken to verify the equivalence of the particle to PFU ratios. A549 cells were infected with each isolate at MOI 2 and cell viability was measured daily (Fig. 1B).

Infection with all nhpAd isolates reduced cell viability to ≤20% after 3–7 days post-infection. Most viruses demonstrated similar (AdV-lumc006, AdV-lumc007, AdV-lumc009, and AdV-lumc010) or slightly delayed (AdV-lumc001 to 005, AdV-lumc008, and AdV-lumc011) cell killing kinetics compared to HAdV-C5. In contrast, AdV-lumc012 demonstrated near-complete cell killing already at 3 days post-infection. Despite the varying kinetics, all nhpAd isolates were well capable of cell killing within a relevant time frame and at a relatively low MOI. Moreover, the nhpAd isolates classified as members of the HAdV-B species (AdV-lumc006, AdV-lumc007, and AdV-lumc012) were similar to HAdV-C5 regarding their ability to kill A549 and were superior to the nhpAd isolates of HAdV-C.

### nhpAds show broad oncolytic potential in human cancer cell lines

**Figure 2.**
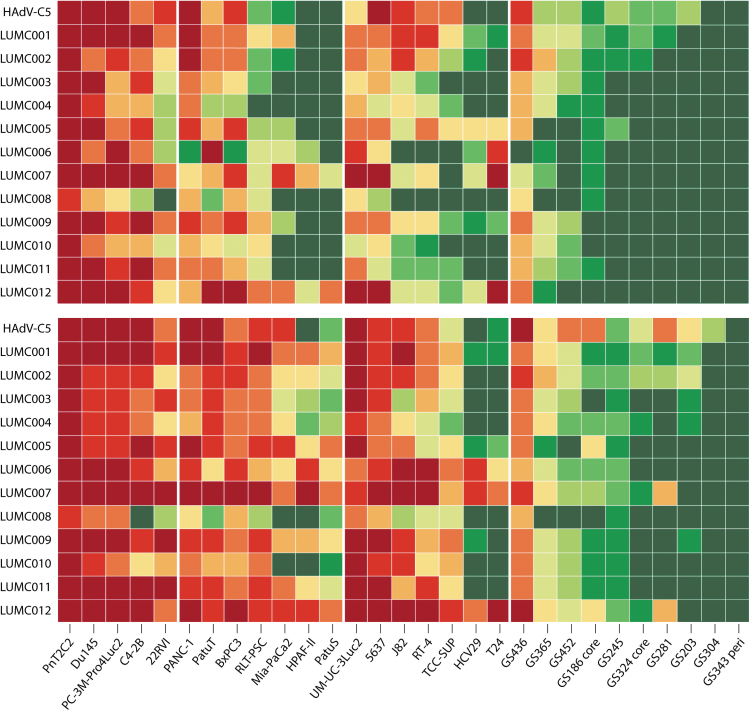
Cell viability of 29 human cancer-associated cell lines after exposure to nhpAds. Human cancer cell lines derived from four cancer types, including—from *left* to *right*—prostate cancer (5), pancreatic cancer (7), bladder cancer (7), and glioblastoma (10), were seeded at 5,000 cells/well in a flat-bottom 96-well plate in cell culture medium one day before infection. Cells were infected with each nhpAd at MOI 25 under low or no serum conditions, depending on the cell line. Cell viability was measured at 3 (*upper panel*) and 5 (*lower panel*) days post-infection. The experiment was performed in triplicate and lytic activity is illustrated in color code ranging from high activity (<10% cell viability, *dark red*) to low activity (≥90% cell viability, *dark green*).

To extend our evaluation of the potential of nhpAds as oncolytic agents, each of the 12 isolates was tested on a broad panel of tumor cell lines derived from aggressive tumor types. Again, wild-type HAdV-C5 was included as a reference. All the nhpAd isolates induced oncolysis in at least a subset of the tumor cell lines, measured as mean cell viability after 3 and 5 days post-infection (Fig. 2). Although there was considerable variability in the susceptibility of different tumor types and cell lines, in general, the prostate cancer cell lines were most sensitive to Ad-induced oncolysis, followed by pancreatic and bladder cancer cell lines. In addition, all nhpAds also showed effective oncolysis of a tumor-associated stroma cell line (RLT-PSC). The glioblastoma cell lines were relatively insensitive to adenoviral infection, apart from GS436, although this may be dependent on the different culture conditions for these cells.

Two bladder cancer cell lines (T24 and HCV29) were selectively killed by AdV-lumc006, AdV-lumc007, and AdV-lumc012. Interestingly, these Ads were all classified as HAdV-B species, as opposed to the remaining Ads in our panel. The hAds of the HAdV-B species are known to make use of more ubiquitously expressed receptors (*i.e.,* the complement receptor CD46) compared to the coxsackie and adenovirus receptor (CAR), the main entry receptor for most other hAd species.^38^ As both T24^39^ and HCV29^40^ have been described to lack the CAR, the difference in susceptibility in these cell lines can be explained by the differential use of cellular receptors for entry into the host cell. Bearing in mind the differences in receptor use, there is still some variability in the oncolytic potency of the different isolates. Overall, AdV-lumc007 and AdV-lumc012 demonstrated the highest potency, while AdV-lumc008 was least potent and did not induce strong oncolytic effects (defined as <20% mean cell viability) in any of the cell lines, except for PnT2C2, a prostate cancer cell line.

It should be noted that the potency of the viruses to induce cell killing could not be attributed to their respective host species, as AdV-lumc007 and AdV-lumc008 are both gorilla-derived Ads (Table 1). Remarkably, two nhpAds (AdV-lumc007 and AdV-lumc012) outperformed HAdV-C5 regarding their ability to induce oncolysis in these cell lines. Furthermore, AdV-lumc007 and AdV-lumc012 appeared to kill faster, as illustrated by the greater reduction in cell viability at 3 days post-infection (most notably in the bladder cancer cell lines). In addition, none of the other nhpAd isolates was able to infect as many cancer cell lines as AdV-lumc007 and AdV-lumc012. In summary, these data indicate that the HAdV-B isolates AdV-lumc007 and AdV-lumc012 could be promising candidates for virotherapy as they can infect and kill a broad spectrum of human cancers.

### nhpAds of HAdV-B show greater genomic resemblance to hAds

To provide some insight in the factors that may underlie the oncolytic potential of these nhpAds, next-generation sequencing was performed on all 12 isolates to determine the nucleotide sequences of the complete viral genomes. All genomes were filed in Genbank and can be assessed by their accession number. The viral genomes varied in length from 35,179 bp (AdV-lumc006) to 37,885 bp (AdV-lumc009, Table 1). Analyses of the genomes confirmed the previously determined classification of nhpAd isolates as either HAdV-B or HAdV-C species based on sequence similarity of the whole genome as well as the conserved DNA polymerase gene (Supplementary Fig. S1). Ads isolated from great apes have frequently been classified within the HAdV species, which derives from their shared ancestry with hAds, recurrent bidirectional cross-species transmission, as well as their ability for recombination across hominid species.^19^ GC content of the majority of nhpAds was slightly higher (data now shown) than reported for the hAds of the HAdV-B or HAdV-C species.^41^ Finally, sequencing did not show any evidence of homologous recombination between the nhpAd genomes and the HAdV-C5 sequences in the HER911 cells on which the viruses were propagated.

**Figure 3.**
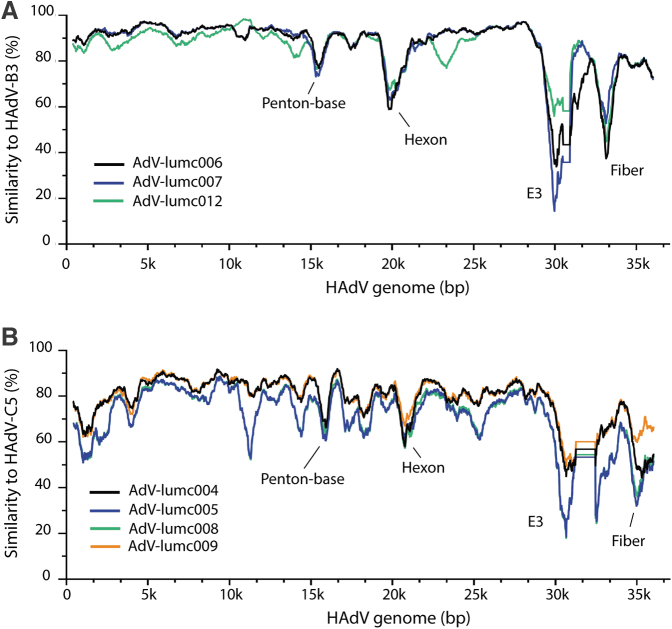
Sequence similarity of nhpAds and hAds of the HAdV-B and HAdV-C species. **(A)** Whole genome sequences of the HAdV-B nhpAds were plotted against HAdV-B3 and **(B)** four HAdV-C nhpAds representing all great ape species were plotted against HAdV-C5. The nhpAds of the HAdV-B species demonstrated an average sequence similarity of ∼86%, while the nhpAds of the HAdV-C species ranged from 70% (AdV-lumc005) to 79% (AdV-lumc009). Indicated are the regions harboring the greatest dissimilarity: *penton-base*, *hexon*, *E3*, and *fiber*. Genome sequences were aligned using MAFFT v.3.8.1551 and plots were generated using SimPlot v3.5.1 with window size 800 and step size 20. Accession numbers: HAdV-C5 (AC_000008.1), HAdV-B3 (NC_011203.1), and the nhpAds (Table 1).

Next, comparative analyses were performed between the nhpAds and HAdV-B3 or HAdV-C5, as human-derived representatives of the respective Ad species (Fig. 3). The HAdV-B nhpAds (AdV-lumc006, AdV-lumc007, and AdV-lumc012) demonstrated high sequence similarity to their human counterpart HAdV-B3 (average similarity ∼86%), with the exception of four gene regions. As expected, the gene regions of high diversity included the major capsid proteins: hexon, fiber, and penton-base. The fourth region, located at ∼28,000 bp to ∼32,000 bp, harbored the greatest variability and covers most of the early region *E3*. The *E3* gene region harbors a series of genes that have immunomodulatory functions and facilitate evasion of T cell and NK-cell recognition of infected cells, as well as reduce sensitivity to (TNF mediated) apoptosis.^42^ Interestingly, this specific region showed higher variability between Ad types derived from human and nonhuman primate origin when compared to hAds alone (data not shown).^43^ For the HAdV-C viruses, average similarity of the nhpAds and HAdV-C5 was slightly lower (∼70–79%) compared to the HAdV-B nhpAds and HAdV-B3. Nevertheless, the highest variability was still observed in the hexon, fiber, penton-base, and *E3* region.

Interestingly, the orangutan-derived AdV-lumc005 showed the least similarity to HAdV-C5. Furthermore, there was less sequence similarity for E1A, and to a lesser extent E1B, between HAdV-C nhpAds and a hAd of the same species compared to the HAdV-B viruses. Considering that E1 is essential for adenoviral replication, variations in this region could have an impact on the replication potential of HAdV-C nhpAds in human cells. Consequently, this might explain some of the differences we observed in the oncolytic potential between the nhpAds of HAdV-B and HAdV-C species (Fig. 2).

### Election of the gorilla-derived AdV-lumc007 for development of an oncolytic Ad

**Figure 4.**
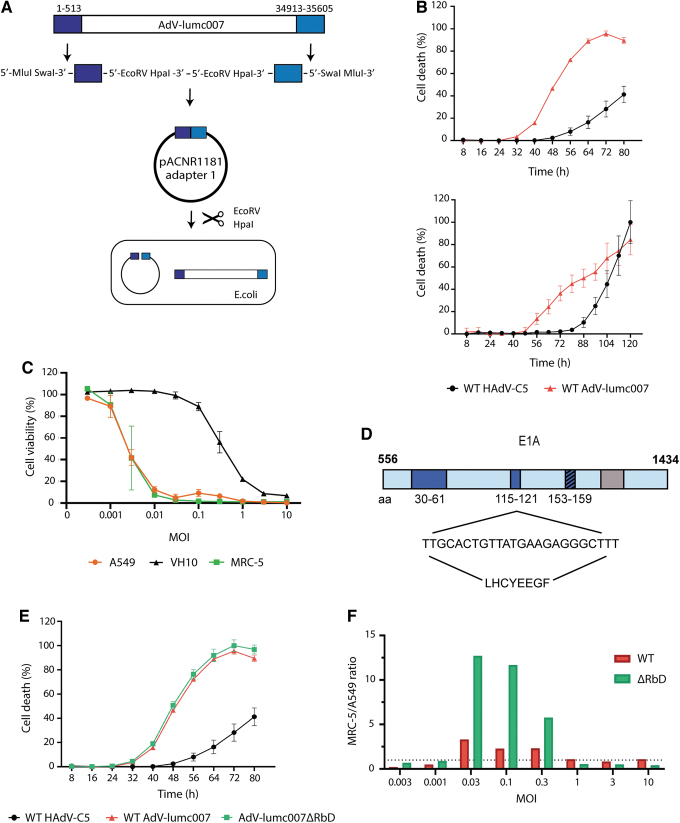
Generation of the gorilla-derived oncolytic adenovirus AdV-lumc007ΔRbD. **(A)** Schematic representation of the generation of the viral vector AdV-lumc007; **(B)** A549 cells were infected with CsCl-purified batches of wt HAdV-C5 or wt AdV-lumc007 at MOI 2 (*upper panel*) or MOI 0.4 (*lower panel*) and cell death was determined every 8 hours using the Incucyte S3 Live-Cell Analysis System. Mean and SD are depicted of a representative figure of three independent experiments, each performed in triplicate; **(C)** A549 cells and the normal fibroblast cell lines VH10 and MRC-5 were infected with wt AdV-lumc007 and cell viability was measured by WST assay at 5 days post-infection. Mean and SD are depicted of a representative figure of three independent experiments, each performed in triplicate; **(D)** putative Rb-binding sites in the AdV-lumc007 *E1A* gene were identified based on sequence homology to HAdV-C5^44,45^; **(E)** A549 cells were infected with WT HAdV-C5, wt AdV-lumc007, or AdV-lumc007ΔRbD at MOI 2 and cell death was determined every 8 hours using the Incucyte S3 Live-Cell Analysis System. Mean and SD are depicted of a representative figure of three independent experiments, each performed in triplicate; **(F)** A549 and MRC-5 cells were infected with wt AdV-lumc007 or AdV-lumc007ΔRbD and cell viability was measured at 5 days post-infection. The MRC-5/A549 ratio was determined by dividing the mean cell viabilities accordingly, where *y* > 1 indicates tumor-selectivity. The mean is depicted of a representative figure of three independent experiments, each performed in triplicate.

As a proof-of-concept, the gorilla-derived HAdV-B AdV-lumc007 was elected for further development as an oncolytic agent as it showed the least variability in the neutralization assay and was shown to be one of the top oncolytic candidates in the screening. A plasmid clone of AdV-lumc007 was generated by synthesizing a double-stranded DNA fragment that encompassed the left-hand (1–513) and right-hand side (34913–35605) nucleotides of the AdV-lumc007 genome flanked by several restriction sites (Fig. 4A). The plasmid was introduced into *E. coli* together with extrachromosomal DNA isolated from AdV-lumc007-infected cells to allow for the generation of a new plasmid containing the entire AdV-lumc007 genome by means of homologous recombination. The plasmid was used to transfect HER911 cells for the generation of new Ad particles, of which a CsCl-purified virus batch was produced.

In accordance with our screening panel, the newly generated gorilla-derived oncolytic AdV-lumc007 was found to infect at an earlier onset and more rapidly (±16 h difference) compared to HAdV-C5 (Fig. 4B, upper panel). This was accompanied by faster replication of viral genomes (Supplementary Fig. S2). Moreover, these differences were retained at lower MOIs, indicating not only faster cell killing but also faster viral dissemination (Fig. 4B, lower panel). Taken together, these data demonstrate that the plasmid clone contains all elements of the AdV-lumc007 virus necessary for efficient replication of this virus.

### Deletion of the E1A Rb-binding domain limits AdV-lumc007′s lytic potential in normal cells

To address the virus' selectivity for tumor cells, tumor cells and normal fibroblasts were infected with wild-type (wt) AdV-lumc007 and cell viability was determined 5 days post-infection (Fig. 4C). Unexpectedly, wt AdV-lumc007 lytic potential in normal cell lines was variable between cell lines. Compared to A549, the fibroblast MRC-5 cell line was similarly susceptible to infection, while the fibroblast VH10 cell line was much less susceptible. We speculated that these differences were related to the varying growth kinetics of the two cell lines, wherein the VH10 cell line was notably slower (data not shown). From this, we hypothesized that we could enhance AdV-lumc007′s dependency on cell proliferation even further by modification of one retinoblastoma (Rb)-binding domain in the *E1A* gene. The Rb protein is an inhibitor of the cell cycle and a master regulator in the progression from G1 to S-phase.^44^ For HAdV-C5, a 24-bp deletion in one Rb-binding domain of *E1A* gene has previously shown to generate a mutant Ad with selective replication in tumor cells.^45^ Therefore, the *E1A* gene of AdV-lumc007 was analyzed for putative Rb-binding sites (Fig. 4D). Three potential sites were identified, of which the second (LHCYEEGF) demonstrated the greatest similarity to the Rb-binding site of HAdV-C5 E1A.

Deletion of eight amino acids was accomplished by IVA cloning^34^ and one of the plasmid clones containing the deletion was used for transfection in HER911 cells together with the original plasmid to generate new virus particles by means of homologous recombination. Viruses were plaque purified and checked for the deletion by sequencing of the *E1A* gene (data not shown). The resulting mutant virus containing the deletion of the Rb-binding domain was named AdV-lumc007ΔRbD or “Goravir.” A purified batch of the newly generated oncolytic derivative demonstrated similar replication potential as its parental virus (Supplementary Fig. S2) and retained its oncolytic potential in A549 cells (Fig. 4E). Finally, both wt AdV-lumc007 and AdV-lumc007ΔRbD were tested for their oncolytic potential in normal cells. As anticipated, the deletion reduced AdV-lumc007′s lytic potential in MRC-5 cells, while retaining its potential in tumorous A549 cells (Fig. 4E, F). Henceforth, it seems that genetic modifications of the hAd genome that increase tumor selectivity can be exploited in nhpAds.

## DISCUSSION

The use of nhpAds for development of new vaccine vectors has shown to be a valuable alternative for the use of hAds. Their success as vaccine vectors as well as their resemblance to hAds have raised the question whether these viruses could also be used as OVs.^19^ In this study, we characterized 12 new nhpAd isolates for their potential as oncolytic agents by assessing the degree of neutralizing immunity against these viruses as well as their oncolytic potential in a variety of human cancer cell lines. Surprisingly, for several nhpAds of the HAdV-C species, the presence of nAbs in pooled IVIg was only moderately lower compared to HAdV-C5 and for none of these isolates, neutralization was absent (Table 2).

The presence of neutralizing activity against these nhpAds, despite their unique HHVR sequences, indicates that other hexon epitopes or other viral capsid proteins are recognized, such as the fiber or penton-base proteins^35,46,47^ or that the nhpAds are susceptible to cross-neutralization by nAb directed against HHVR-epitopes raised against hAds circulating in the human population. In support of the latter, Paris *et al.* showed that vaccination with HAdV-E4 elicited heterotypic neutralizing immunity to both human and nhpAds.^37^ Most importantly, the largest response was not seen for the HAdV-E species but for the chimpanzee-derived HAdV-C species ChAd3. Therefore, nhpAds of the HAdV-C species might be particularly susceptible to cross-neutralization.

Interestingly, nhpAds of the HAdV-B species were not neutralized, suggesting that they are not affected by neutralization as a result of cross-reactivity. In addition, the presence of nAbs did not seem to depend on the host from which the Ad was isolated, as we were unable to detect nAbs against the HAdV-B AdV-lumc006 and AdV-lumc007 (both gorilla), as well as AdV-lumc012 (chimpanzee). In light of these data, it can be expected that these nhpAds are also not cross-reactive with antibodies against current vaccines based on nhpAds, like the chimpanzee-Ad Covid vaccine produced by AstraZeneca.

The potential of the HAdV-B nhpAds was further highlighted when the nhpAds were tested for their oncolytic abilities in human cancer cells. While all nhpAd isolates demonstrated efficient cell killing of at least a subset of human cancer cell lines, the nhpAds of the HAdV-B species showed oncolytic activity in a broad range of cancer cell lines (Fig. 2). So far, this ability of the HAdV-B nhpAds remains unique, as the generation of an oncolytic vector from an nhpAd of the HAdV-E species demonstrated only moderate cytotoxicity in a limited number of cancer cell lines.^48^ The use of a relatively high MOI in our assay may have favored the selection of a cytotoxic virus variant rather than a variant that produces high viral titers. However, as the nhpAd isolates of the HAdV-B and HAdV-C species behave rather similarly to each other in their respective group, we do not expect other isolates to excel upon the use of different conditions. One possible explanation for the observed difference between the Ad species might be related to their genomic similarity to hAds of the same species, especially in gene regions involved in perturbation of the cell cycle (Fig. 3).

Interestingly, the nhpAds of the HAdV-C species demonstrated greater variability in the E1 region compared to hAds of the same species than nhpAds of the HAdV-B species did. For E1A alone, there are many functions related to the establishment of a productive infection.^49–51^ Sequence analysis of E1A among 34 different Ad types has previously shown that several binding motifs are conserved across human and nhpAds from different Ad species, while others are not.^52^ As such, variations in these regions might provide an explanation for the variable oncolytic potential.

The differences in lytic efficiency of nhpAds could not depend on the host from which the virus was isolated as illustrated by the gorilla-derived viruses AdV-lumc007 and AdV-lumc008. Taken together, this suggests that neither the varying efficiencies of the nhpAds to infect human cells nor pre-existing neutralizing immunity in the human population correlates with the host species from which the virus was isolated, but rather correlates to the Ad species. Therefore, it seems that nhpAds derived from great apes, especially those of the HAdV-B species, may be exploited as an alternative source for the development of oncolytic adenoviral vectors.

As a proof of concept, we used AdV-lumc007, a gorilla-derived HAdV-B Ad, for the development of an oncolytic agent. This virus demonstrated rapid cell killing and viral dissemination (Fig. 4A, B). Moreover, AdV-lumc007 showed faster kinetics than HAdV-C5 and was able to infect a broader range of cancer cells. The broad infection range may relate to the receptor used by this virus as many HAdV-B types can use the CD46 protein as their entry receptor. In addition to being ubiquitously expressed, this protein is upregulated in many tumor types.^53^ This has favored the development of several oncolytic derivatives from hAds of the HAdV-B species, including HAdV-B3,^54^ HAdV-B11,^55^ and more recently HAdV-B35.^56^ While seroprevalence varies between these Ad types, the reported cytotoxicity of these viruses often does not surpass that of HAdV-C5. However, only parallel testing of multiple oncolytic Ads derived from different HAdV types would allow fair quantitative comparisons.

AdV-lumc007 demonstrated some degree of tumor selectivity, although this was rather cell line dependent. Ads are not known to be inherently tumor-selective, although previously, a bioselected hybrid HAdV-B hAd demonstrated an enhanced capacity to replicate in tumor cells. This virus, named Enadenotucirev (EnAd, formerly known as ColoAd1), is a hybrid between the HAdV-B3 and HAdV-B11p, where the capsid proteins are derived from the HAdV-B11p.^55^ Interestingly, EnAd has a 25nt deletion in the *E4orf4* region, resulting in a frame-shift. The impact of this mutation on oncolytic activity remains to be established, but given its location, it could be hypothesized that this deletion attenuated the virus resulting in its improved tumor-selectivity. However, we have not detected such alterations in AdV-lumc007. Alternatively, we deleted the E1A Rb-binding domain as described in Suzuki *et al.*^57^, thereby selectively limiting AdV-lumc007′s lytic potential in normal cells (Fig. 4F).

Taken from this, it seems that the overall high sequence similarity of nhpAds to hAds, for the HAdV-B species especially, allows for the genetic modification of nhpAds using well-established modifications of clinically applied hAd-derived viruses.^45,58^ In support of this, a recent study showed that another nhpAd named Gad (formerly GC46) was amendable to capsid modifications similar to HAdV-C5.^59^ The ease by which nhpAd-derived vectors can be generated by building upon the expertise gathered previously using hAd vectors provides a solid foundation for the development of such vectors.

## CONCLUSION

In conclusion, we are the first to have successfully generated a gorilla-derived oncolytic Ad, which seems to have low seroprevalence in the human population. The oncolytic activity of AdV-lumc007 and Goravir was often similar, and in several cell systems even superior, to HAdV-C5. While the virus has not yet been tested *in vivo*, the *in vitro* data seem promising regarding future clinical development. Established pre-clinical models derived from resected tumor material such as organoid^60^ and tumor-slice^61^ cultures may provide further insights into Goravir's antitumor efficacy.

However, its success is not merely defined by its oncolytic potential. The efficacy of oncolytic virotherapy is largely dependent on the ability to mediate antitumor immune responses. As such combining OVs with immunomodulatory drugs seems a promising approach.^62^ However, a complete understanding of the mechanism of action of the viruses is often lacking, which hampers optimization of these viruses for treatment.^63^ For hAds, there are considerable differences between Ad types regarding their activation of cell death pathways and modulation of immune responses,^3,6,64^ both of which are important to mount effective antitumor responses. Therefore, it seems essential to acquire such knowledge on nhpAds in general and on Goravir in particular, to allow for the design of new treatment regimens that patients need.

## Supplementary Material

Supplemental data

Supplemental data

Supplemental data
