# Supplementary material for: Nonhuman Primate Adenoviruses of the Human Adenovirus B Species Are Potent and Broadly Acting Oncolytic Vector Candidates
Source: Hum Gene Ther. 2022 Mar 16;33(5-6):275–89. doi: 10.1089/hum.2021.216 (PMC8972008; doi:10.1089/hum.2021.216)
Supplement: Supplemental data [file Supp_TableS1.pdf]

Supplementary Table 1. Recovery of non-human primate (nhp) adenoviruses (Ads) from stool samples of great apes held in captivity.

	Bonobo	Chimpanzee	Gorilla	Orangutan	Total
**CPE+**	8	28	13	3	52
**PCR+**	8	28	13	2	51
**Unique**	6	24	10	1	41
**HAdV-B**	0/6 (0%)	3/24 (12%)	3/10 (30%)	0/1 (0%)	6/41 (15%)
**HAdV-C**	6/6 (100%)	17/24 (71%)	5/10 (50%)	1/1 (100%)	29/41 (70%)
**HAdV-E**	0/0 (0%)	4/24 (17%)	2/10 (20%)	0/1 (0%)	6/41 (15%)
