# Supplementary material for: Nonhuman Primate Adenoviruses of the Human Adenovirus B Species Are Potent and Broadly Acting Oncolytic Vector Candidates
Source: Hum Gene Ther. 2022 Mar 16;33(5-6):275–89. doi: 10.1089/hum.2021.216 (PMC8972008; doi:10.1089/hum.2021.216)
Supplement: Supplemental data [file Supp_FigS1.pdf]

A

B

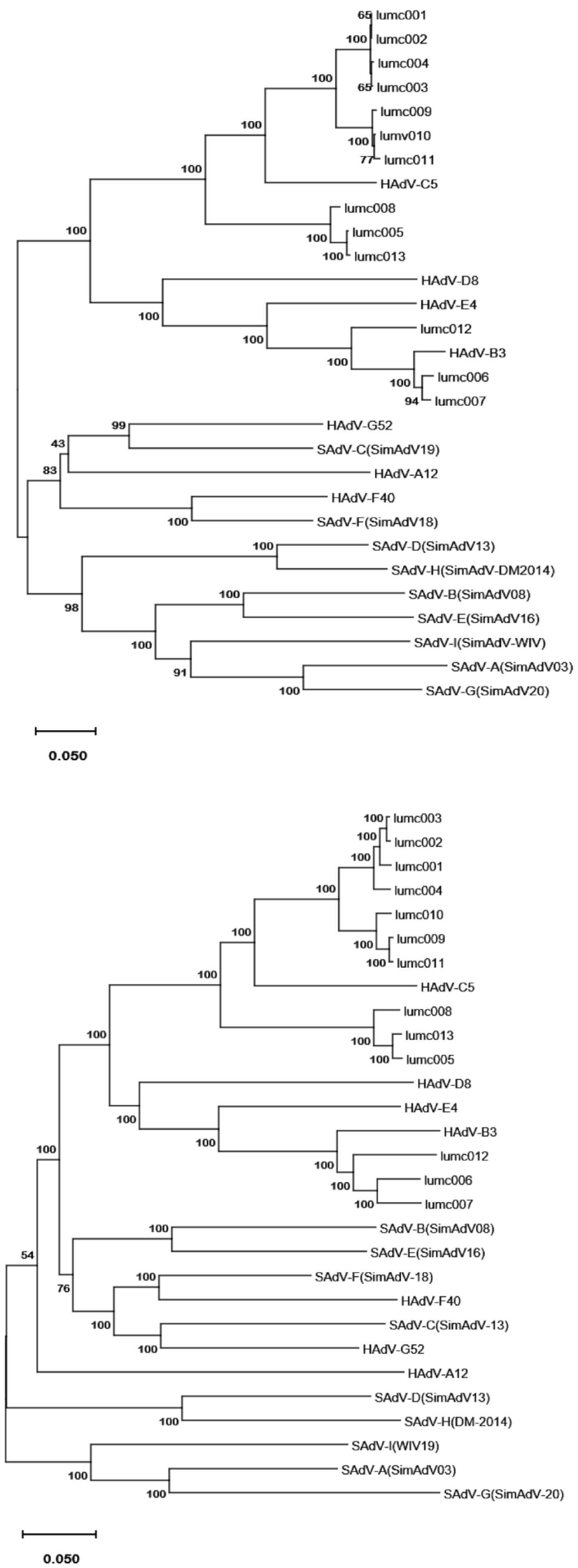

lumc001
lumc002
lumc004
lumc003
lumc009
lumv010
lumc011
HAdV-C5
lumc008
lumc005
lumc013
HAdV-D8
HAdV-E4
lumc012
HAdV-B3
lumc006
lumc007
HAdV-G52
SAdV-C(SimAdV19)
HAdV-A12
HAdV-F40
SAdV-F(SimAdV18)
SAdV-D(SimAdV13)
SAdV-H(SimAdV-DM2014)
SAdV-B(SimAdV08)
SAdV-E(SimAdV16)
SAdV-I(SimAdV-WIV)
SAdV-A(SimAdV03)
SAdV-G(SimAdV20)
0.050
lumc003
lumc002
lumc001
lumc004
lumc010
lumc009
lumc011
HAdV-C5
lumc008
lumc013
lumc005
HAdV-D8
HAdV-E4
HAdV-B3
lumc012
lumc006
lumc007
SAdV-B(SimAdV08)
SAdV-E(SimAdV16)
SAdV-F(SimAdV-18)
HAdV-F40
SAdV-C(SimAdV-13)
HAdV-G52
HAdV-A12
SAdV-D(SimAdV13)
SAdV-H(DM-2014)
SAdV-I(WIV19)
SAdV-A(SimAdV03)
SAdV-G(SimAdV-20)
0.050

**Supplementary Figure S1. Evolutionary relationships of the nhpAd isolates.** The evolutionary history was inferred using the Neighbor-Joining method[62] after aligning the sequences with MAFFT version 7.427[63]. The optimal tree is shown. The percentage of replicate trees in which the associated taxa clustered together in the bootstrap test (500 replicates) are shown next to the branches[64]. The tree is drawn to scale, with branch lengths in the same units as those of the evolutionary distances used to infer the phylogenetic tree. The evolutionary distances were computed using the Poisson correction method[65] and are in the units of the number of amino acid substitutions per site. This analysis involved 29 amino acid sequences of A) whole genome sequences, or B) DNA binding protein (DBP). All ambiguous positions were removed for each sequence pair (pairwise deletion option). There were a total of 632 positions in the final dataset. Evolutionary analyses were conducted in MEGA X[66]. Accession numbers: HAdV-A12 (X73487), HAdV-B3 (NC_011203), HAdV-C5 (AC_000008), HAdV-D8 (KP016723), HAdV-E4 (AY458656), HAdV-F40 (KU162869), HAdV-G52 (DQ923122), SimAdV-19 (NC_028107), SimAdV-18 (FJ025931), SimAdV-13 (NC_028103), SimAdV-DM2014 (NC_025678), SimAdV-08 (NC_028113), SimAdV-16 (NC_028105), SimAdV-WIV19 (KX505867), SimAdV-03 (NC_006144), and SimAdV-20 (NC_020485).
