# Supplementary material for: Nonhuman Primate Adenoviruses of the Human Adenovirus B Species Are Potent and Broadly Acting Oncolytic Vector Candidates
Source: Hum Gene Ther. 2022 Mar 16;33(5-6):275–89. doi: 10.1089/hum.2021.216 (PMC8972008; doi:10.1089/hum.2021.216)
Supplement: Supplemental data [file Supp_FigS2.pdf]

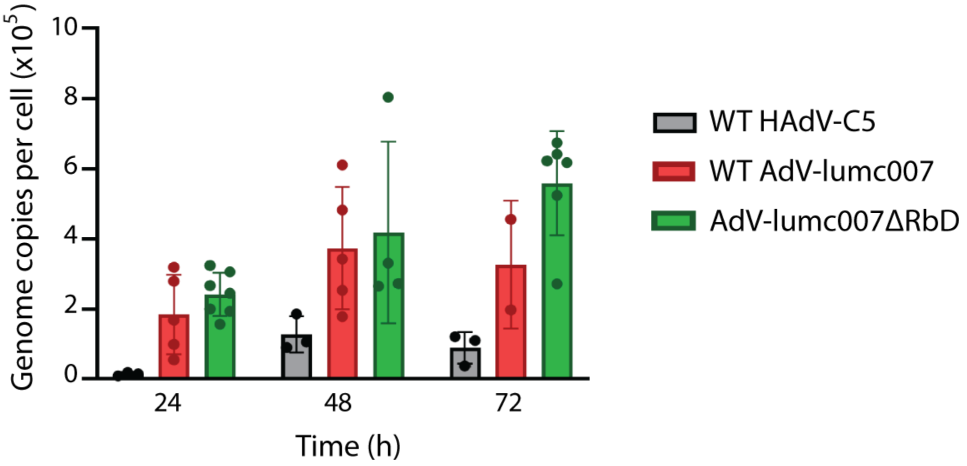

Supplementary Figure S2. Virus replication of HAdV-C5, WT AdV-lumc007, and AdV-lumc007ΔRbD in A549 cells. A549 cells were infected with virus at MOI 2 and cells were collected at 24, 48, and 72 hours post infection. Viral genome copies were determined by qPCR on genomic DNA isolated from the infected cells. Values were normalized to a plasmid standard curve. Dots represent biologically independent replicates. Mean and SD are depicted.
